# Targeting upregulated RNA binding protein RCAN1.1: a promising strategy for neuroprotection in acute ischemic stroke

**DOI:** 10.1111/cns.13921

**Published:** 2022-07-28

**Authors:** Yan Yun, Xiaxin Yang, Shichuan Tan, Pin Wang, Yanbin Ji, Xiulian Sun

**Affiliations:** ^1^ Department of Radiology Qilu Hospital of Shandong University Jinan China; ^2^ Department of Neurology Qilu Hospital of Shandong University Jinan China; ^3^ NHC Key Laboratory of Otorhinolaryngology Qilu Hospital of Shandong University Jinan China; ^4^ Department of Otorhinolaryngology Qilu Hospital of Shandong University Jinan China; ^5^ Brain Research Institute Qilu Hospital of Shandong University Jinan China; ^6^ The Key Laboratory of Cardiovascular Remodeling and Function Research, Chinese Ministry of Education, Chinese National Health Commission Qilu Hospital of Shandong University Jinan China

**Keywords:** acute ischemic stroke, apoptosis, HIF1α, R1SR13, RBP, RCAN1

## Abstract

**Aims:**

To explore the expression changes and roles of the RNA‐binding protein RCAN1.1 in acute ischemic stroke (AIS), and to preliminarily confirm the medicinal value of the RNA aptamer R1SR13 in AIS by targeting RCAN1.1.

**Methods:**

Two mouse AIS models of middle cerebral artery occlusion (MCAO) and right common carotid artery ligation (R‐CCAL) and oxygen glucose deprivation (OGD) model of AIS in primary neurons and SH‐SY5Y were performed. The expression pattern of RCAN1.1 was assessed using real‐time quantitative PCR (RT‐qPCR) and western blotting (WB) in vivo and in vitro. The underlying mechanism for the elevation of RCAN1.1 in the upstream was investigated. Lentiviruses were administrated and the effect of RCAN1.1 in AIS was assessed by ATP level, caspase 3/7 assay, TUNEL and WB. The protective function of R1SR13 in AIS was evaluated both in vivo and in vitro.

**Results:**

In two mouse models of AIS, *RCAN1.1* mRNA and RCAN1.1 L protein were significantly upregulated in the ischemic brain tissue. The same results were detected in the OGD model of primary neurons and SH‐SY5Y. The mechanistic analysis proved that hypoxia‐inducible factor‐1α (HIF1α) could specifically activate the *RCAN1.1* gene promoter through combining with the functional hypoxia‐responsive element (HRE) site (−325 to −322 bp). The increased expression of RCAN1.1 L markedly depleted ATP production and aggravated neuronal apoptosis under OGD condition. R1SR13, an antagonizing RNA aptamer of RCAN1.1, was demonstrated to reduce neuronal apoptosis caused by the elevated RCAN1.1 L in the cellular and animal models of AIS.

**Conclusion:**

*RCAN1.1* is a novel target gene of HIF1α and the functional HRE in the RCAN1.1 promoter region is −325 to −322 bp. The marked upregulation of RCAN1.1 in AIS promoted neuronal apoptosis, an effect that could be reversed by its RNA aptamer R1SR13 in vivo and in vitro. Thus, R1SR13 represents a promising strategy for neuroprotection in AIS and our study lays a theoretical foundation for it to become a clinically targeted drug.

## Graphical abstract

The study demonstrates that the upregulation of RCAN1.1 by HIF1α in acute ischemic stroke promotes neuronal apoptosis, an effect that could be reversed by its RNA aptamer R1SR13. Thus, R1SR13 represents a promising strategy for neuroprotection in acute ischemic stroke and the study lays a theoretical foundation for it to become a clinically targeted drug.

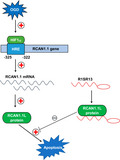

## 1 INTRODUCTION

Stroke represents one of the leading causes of mortality and morbidity in China and even across the globe. Nearly 80% of acute stroke scenarios are classified as ischemic, as the consequence of sudden obstruction of criminal arteries, leading to the ischemia and hypoxia of involved brain tissue.^1^, ^2^, ^3^ The timely restoration of blood supply in the acute phase is the most efficacious therapeutic strategy in contemporary clinical practice of acute ischemic stroke (AIS). However, the narrow therapeutic window and side effects severely restrict clinical application. Despite intensive researches carried out on the neuroprotective agents in recent years, unfortunately, none has been validated as beneficial in clinical application.^1^, ^4^, ^5^ Therefore, a thorough understanding of the underlying mechanisms about AIS becomes urgent, and a more potent treatment scheme is badly needed.

The Regulator of calcineurin 1 gene (*RCAN1*) was first identified as the Down Syndrome critical region 1 (*DSCR1*) gene on chromosome 21q22, consisting of seven exons and six introns.^6^ The first four exons are alternatively sliced to generate four transcripts, and the RCAN1 isoform 1 (RCAN1.1) is a major one highly expressed in the brain.^7^ There are two different isoforms of RCAN1.1 as a consequence of two distinct translational start codons (AUG), a full‐length isoform (RCAN1.1 L) and a shorter isoform (RCAN1.1S). The full‐length isoform, RCAN1.1 L, contains the sequence of the shorter isoform (RCAN1.1S), leading to the identical function of the two isoforms.^8^

RCAN1.1 proteins are highly conservative and multifunctional. RCAN1.1 inhibits calcineurin A and calcineurin‐dependent NFAT signaling pathway.^9^, ^10^, ^11^ Our previous studies proved that RCAN1.1 could inhibit NF‐κB signaling pathway via IκBα phosphorylation.^12^, ^13^ Long‐term accumulation of RCAN1.1 L facilitates cell apoptosis through caspase‐3 activation.^8^, ^14^ RCAN1.1S increases adenine nucleotide translocator 1(ANT1) and leads to mitochondrial dysfunctions.^15^ Wong et al.^16^ also proved that RCAN1.1S overexpression promoted age‐dependent mitochondrial dysregulation in mice brain. In addition, we also found that RCAN1.1 could stimulate N‐glycosylation of BACE1 and NCT in the endoplasmic reticulum.^17^

RNA‐binding proteins (RBPs), interacting with multiple RNA species and 100 of individual transcripts, are of fundamental importance for post‐transcriptional gene regulation and protein synthesis.^18^ Our previous studies have demonstrated that RCAN1.1 is a novel RNA‐binding protein, binding to 269–291 nt of *ANT1* mRNA.^15^, ^19^ ANT1 is the most abundant protein in the inner mitochondrial membrane and has regulatory role in mitochondria‐mediated apoptosis. Overexpression of RCAN1.1S significantly increased the expression of ANT1 by stabilizing *ANT1* mRNA, leading to accelerated ATP‐ADP exchange rate, more Ca_2_ + −induced mitochondrial permeability transition pore (mPTP) opening, increased cytochrome c (Cyt c) release and, eventually, cell apoptosis.^15^

The RNA aptamer of RCAN1.1, R1SR13 was firstly identified by SELEX analysis which had high binding affinity with RCAN1.1 protein. Aptamers are relatively easily screened, synthesized, programmably designed, and chemically modified for various biomedical applications, including targeted therapy.^20^ We had proved that R1SR13 aptamer could inhibit RCAN1.1‐mediated NFAT and NF‐κB‐signaling pathways.^19^ However, the effects of RCAN1.1 and R1SR13 aptamer on cerebral ischemic stroke have not been thoroughly investigated.

In the current study, we therefore characterized the changes of RCAN1.1 protein expression after AIS, both in vivo and in vitro. We discovered that *RCAN1.1* transcript and RCAN1.1 L protein was significantly upregulated in AIS both in vivo and in vitro and HIF1α activated RCAN1.1 expression through binding to its promoter. Overexpression of RCAN1.1 L significantly increased cell apoptosis under OGD condition, while knockdown of RCAN1.1 sharply decreased OGD‐induced cell apoptosis. Furthermore, we found that the RNA aptamer of RCAN1.1, R1SR13 attenuated RCAN1.1‐induced neuronal apoptosis both in vivo and in vitro. Our work proved that overexpression of RCAN1.1 by HIF1α aggravates neuronal apoptosis in AIS and the RNA aptamer of RCAN1.1, R1SR13 has a neuroprotective effect in AIS.

## 2 MATERIALS AND METHODS

### 2.1 Cell culture

The human neuroblastoma SH‐SY5Y cells were obtained from the ATCC and cultured in the Dulbecco's Modified Eagle's Medium (DMEM, high glucose) supplemented with 10% (vol/vol) fetal bovine serum (FBS) and 100 units/ml penicillin and 0.1 mg/ml streptomycin. The primary neurons were obtained from the embryos of E18 Sprague Dawley pregnant rats and cultured as previously described.^14^ The Sprague Dawley rats were from the Experimental Animal Center of Shandong University. All the cultured cells were maintained in a 37°C incubator containing 5% CO_2_.

### 2.2 Oxygen glucose deprivation (OGD) treatment in the cultured cells

The SH‐SY5Y cells and primary neurons were washed twice with the phosphate‐buffered saline (PBS, pH 7.4) and then refreshed with the glucose‐free DMEM (Thermo). The experimental groups were then placed in an anaerobic chamber with 1% O_2_ for the indicated time (0, 2, 4, 6, 8 and 10 h) at 37°C. The control cells were incubated with the complete DMEM in the control condition as described above for the same periods of time.

### 2.3 Cerebral ischemic stroke model of mice

The male C57BL/6 mice were purchased from the Experimental Animal Center of Shandong University at the age of 8 weeks weighing from 25 to 30 g. All the procedures were approved by the Institutional Animal Care and Use Committee of Shandong University, according to the guidelines of the National Institutes of Health on the care and use of animals. Permanent MCAO model was performed to induce a focal cerebral ischemic stroke as previously described.^21^ Briefly, mice were anesthetized with intraperitoneal injection of pentobarbital sodium. Under the dissecting microscope, the right common carotid artery (CCA) and external carotid artery (ECA) were isolated. A piece of monofilament nylon suture with a heat‐rounded tip (BEIJING CINONTECH CO. LTD) was inserted into ECA and arranged to obstruct the origin of MCA. Successful occlusion was confirmed by the Laser Speckle Contrast Imaging (LSCI) and 2, 3, 5‐triphenyltetrazolium chloride (TTC) staining. The Sham group underwent all surgical procedures but without the suture insertion. Tissue samples dissected from the ischemic penumbra around the occluded MCA were harvested and stored for the following protein extracts and RNA preparation after 24 h of MCAO to detect the expression of RCAN1.1 L. The right common carotid artery ligation (R‐CCAL) of mice was operated for the indicated times (0, 2, 4 and 6 h, the ischemic side). The left common carotid artery was stimulated but not ligated (the contralateral side). The hippocampal CA1 regions of mice were harvested to detect the localized expression levels of RCAN1.1.

### 2.4 Plasmid construction and transfection

The HIF1α expressing plasmid pHA‐HIF1α, the RCAN1.1 expressing plasmids pcDNA3.1‐RCAN1.1 L (no tag) and pcDNA3.1‐RCAN1.1S (no tag) were constructed as previously described.^12^, ^22^ The fragment of 5’upstream region of RCAN1.1 was amplified by PCR with the primers (5’–CCGCTCGAGGTCCTCTTATTTTTCCGCTATTTC–3’ and 5’–CACAAGCTTGTCCTGCAGGTCCACCTC–3’) and cloned into the pGL3‐basic vector to construct the pRCAN1.1 luc‐Long plasmid. The truncated pRCAN1.1 luc‐B, ‐C, and ‐D plasmids were created from the pRCAN1.1 luc‐Long plasmid using the restriction enzymes as previously described.^14^ All the constructed plasmids were confirmed by the DNA sequencing. The transient transfections were performed with the lipofectamine 2000 transfection reagent (Invitrogen) according to the manufacturer's instructions.

### 2.5 Viruses and infection

The recombinant lentiviruses Lenti‐RCAN1.1 L‐GFP, Lenti‐siRCAN1.1‐GFP, Lenti‐R1SR13‐GFP and their control viruses were purchased from the Vigene Bio. Inc. The cells were infected at the concentration of 1 MOI. The rat primary neurons were infected in vitro 5 days after culture. The infectious medium was replaced with the fresh medium within 24 h and incubated for another 5 days before further experiments.

The recombinant adeno‐associated virus 9 (AAV9) vectors expressing R1SR13 (AAV9‐R1SR13) and the control virus (AAV9‐R‐CON) were constructed by the Vigene Bio. Inc. (Shandong, China). The male C57BL/6 mice were anesthetized and injected with 4 ul of AAV9‐R1SR13 or AAV9‐R‐CON (1.5 × 10^12^ μg/ml) at a rate of 0.2 μl/min by the stereotaxic apparatus. The coordinates were adjusted according to the previous report^23^ as follows: point 1, 0.3 mm anterior to the bregma, 3 mm lateral to the midline, 2 mm ventral to the dura; point 2, 1.9 mm posterior to the bregma, 3 mm lateral to the midline, 2 mm ventral to the dura. The permanent MCAO surgery was performed after 3 weeks of the injection.

### 2.6 Neurological function and infarct volume assessment

The 5‐tiered scoring system was conducted by a blinded observer for the neurological assessment after 24 h of MCAO. The following graded scale was applied: 0, normal function; 1, flexion of the torso and contralateral forelimb on lifting the animal by the tail; 2, circling to the contralateral side but normal posture at rest; 3, reclination to the contralateral side at rest; 4, absence of spontaneous motor activity.

The 2,3,5‐triphenyltetrazolium chloride (TTC) staining was performed after 24 h of MCAO to confirm the MCAO model of AIS. Infarction volume was measured by digital camera and analyzed by Image J. The infarct area was calculated across each section and was presented as a percentage relative to the area of the contralateral hemisphere.

### 2.7 siRNA Assay

To confirm the efficiency of HIF1α siRNA, the HIF1α siRNA (siHIF1α) or its negative control (siCON) was transfected into the SH‐SY5Y cells with lipofectamine 3000 transfection reagent. The cells were harvested 48 h after transfection for detecting the protein expression levels of HIF1α. The sequences of siHIF1α were 5’–CCAGCAGACUCAAAUACAATT–3’ and 5’–UUGUAUUUGAGUCUGCUGGTT–3’. The pRCAN1.1 luc‐Long plasmid were co‐transfected with the siHIF1α or siCON into the SH‐SY5Y cells using the Lipofectamine 3000 in accordance with the manufacturer's instructions. After 48 h of the transfection, the cells were exposed to the OGD treatment, or control condition, for 6 h for dual‐luciferase assay.

### 2.8 Dual‐luciferase Assay

The dual luciferase assay was performed 48 h after transfection by a dual‐luciferase reporter assay system (Promega) as previously described.^24^

### 2.9 Electrophoretic mobility shift assay (EMSA) and chromatin immunoprecipitation (ChIP)

The EMSA and ChIP were performed as described previously.^25^ The sense sequences of RCAN1.1‐HRE, RCAN1.1‐HRE mut, consensus HIF1α‐HRE and HIF1α‐HRE mut oligonucleotides were 5’–TGGGAGGCCGTGTCGCTGGG–3’, 5’–TGGGAGGCAAAGTCGCTGGG–3’, 5′–AGCTTGCCCTACGTGCTGTCTCAGA–3′, and 5′–AGCTTGCCCTAAAAGCTGTCTCAG A–3′, respectively. RCAN1.1‐HRE and HIF1α‐HRE oligonucleotides were both end‐labeled with IRDye 800 to generate the double‐stranded probes. The binding of HIF1α with RCAN1.1 promoter in nuclear extraction was confirmed using chromatin immunoprecipitation assay kit (#17–371, Millipore) following the manufacturer's protocol. The SH‐SY5Y cells transfected with the HIF1α expression plasmid pHA‐HIF1α were cross‐linked by formaldehyde (final concentration of 1%) for 10 min at 37°C, and then washed with cold PBS twice. The cells were lysed by 1% SDS lysis buffer and then sheared by sonication. The protein and DNA compounds were pulled down with the HIF1α monoclonal antibody (sc‐13,515, Santa Cruz Biotechnology). The positive and negative controls were anti‐RNA Polymerase II and normal mouse IgG. The primers for ChIP‐PCR were 5′–CGGCCTTAAAGGGGCCAC–3′ and 5′–TGTCAGCAGTCTCCCAGCG–3′.

### 2.10 Real‐time quantitative PCR (RT‐qPCR)

Total RNA was extracted from cells and brain tissues using a Trizol reagent (Cat#15596018, Thermo Fisher Scientific). The real‐time amplification was achieved using the ABI 7900HT Fast real‐time PCR system (Applied Biosystems). The taqMan probes for RCAN1.1 were obtained from Applied Biosystems (TaqMan Gene Expression Assays).

### 2.11 Western blotting and antibodies

The western blotting analysis was performed as described previously.^26^ The primary antibodies were against HIF1α (sc‐13,515, Santa Cruz Biotechnology), RCAN1(DCT3), β‐actin (A5441, Sigma‐Aldrich), cleaved caspase‐3 (#9661, CST) according to the manufacturer's instructions. The detection and quantification were achieved using the Li‐Cor Odyssey imaging system and the software Image J.

### 2.12 ATP and caspase 3/7 assays

The CellTiter‐Glo® 2.0 Assay (G9241, Promega) and Caspase‐Glo® 3/7 Assay (G8091, Promega) were used to detect the ATP levels and caspase 3/7 activities following the manufacturer's protocol. The SH‐SY5Y cells and primary neurons were plated into a 48‐well plate and infected with the lentiviruses for 72 h. The cells were then stimulated by the OGD treatment for the indicated time and harvested with the 100 μl Passive Lysis Buffer (E1941, Promega). The mixture of cell lysis with Caspase 3/7 reagent or CellTiter reagent was incubated at room temperature in a plate shaker (1 h for Caspase 3/7 reagent and 10 min for CellTiter reagent). The Varioskan Flash multimode microplate reader (Thermo Fisher Scientific) was used to detect the luminescence.

### 2.13 TUNEL staining

The TUNEL staining was performed with the In Situ Cell Death Detection Kit (12,156,792,910) according to the manufacturers' instructions. The images were captured by the LSM 880 fluorescent microscope (Carl Zeiss) and analyzed with the image j software.

### 2.14 Data Analysis

The data are presented as means ± SEM from three to four independent experiments. Datasets were tested for normality of distribution with the Shapiro–Wilk test. For normal distribution, the differences between the two groups were evaluated by the Student's *t* test, and those among more than two groups were assessed with one‐way or two‐way anova with Bonferronis multiple comparisons post hoc test. Mann–Whitney U test was used for non‐normally distributed data. The differences were considered statistically significant when *p* value is less than 0.05. All the analyses were performed with the prism software (graphpad Software, Inc.).

## 3 RESULTS

### 3.1 RCAN1.1 is upregulated in response to AIS in vivo and in vitro

**FIGURE 1:**
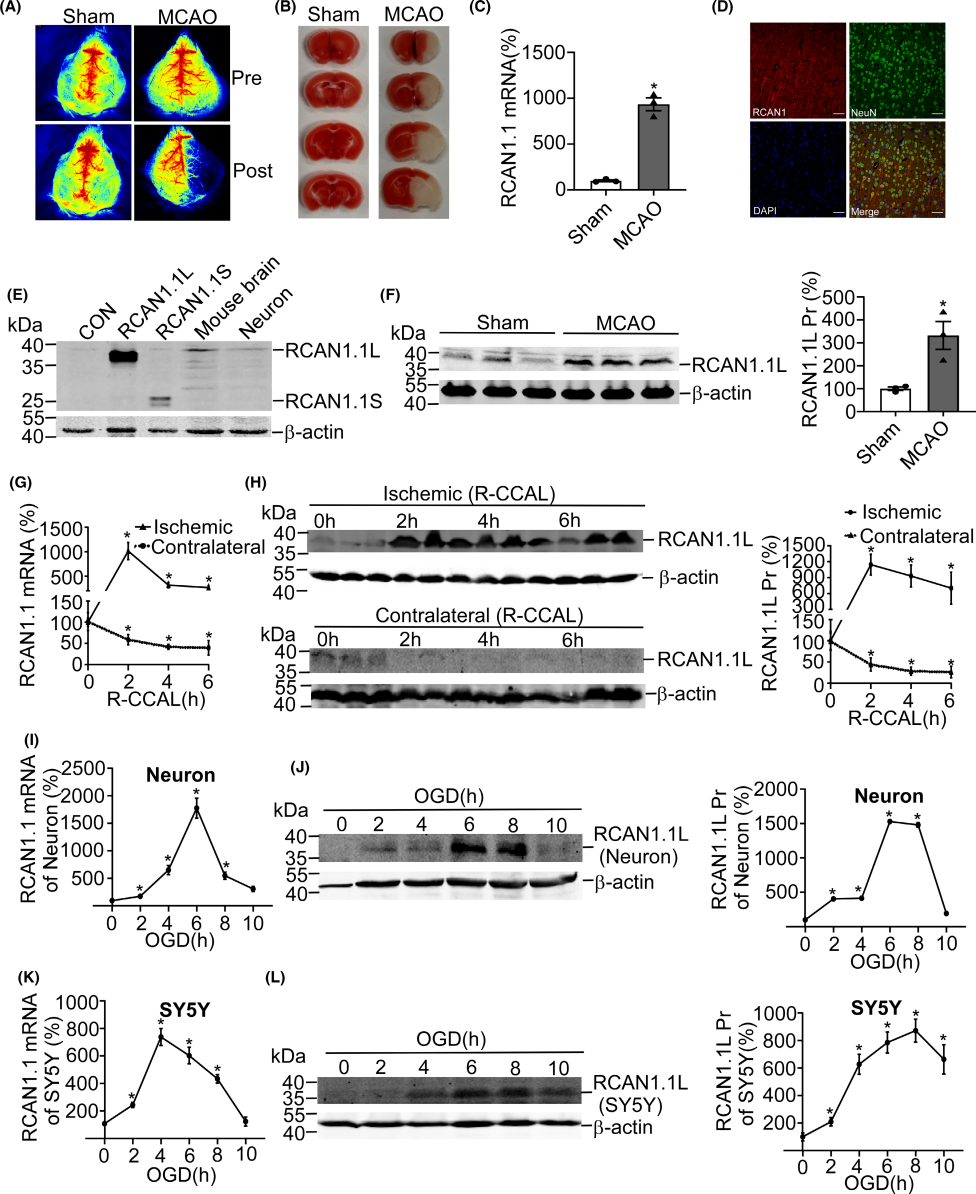
The expression of RCAN1.1 was upregulated in the animal and cellular models of acute ischemic stroke. (A, B) C57BL/6 male mice of 8‐week‐old were performed with MCAO surgery and the laser speckle contrast imaging (LSCI) (A) and TTC staining (B) were performed to confirm the MCAO model of acute ischemic stroke. (C) The mRNA levels of RCAN1.1 were determined in the brain tissues from the MCAO group and the Sham group. Quantified results were acquired from three independent experiments. (D) Immunofluorescence of RCAN1.1 in mouse brain. Double immunofluorescence of RCAN1.1 (red) and NeuN (neuron marker, green) was performed. Images were captured with an LSM880 fluorescence confocal microscope. Scale bars: 50 μm. (E) To investigate the distribution of RCAN1.1 in the mouse brain, HEK293 cells were transfected with pcDNA4‐myc‐His (CON, lane1), pcDNA4‐RCAN1.1 L (no tag, lane2) and pcDNA4‐RCAN1.1S (no tag, lane3) expression plasmids, respectively. Cells were harvested after 48 h and subjected to western blotting using DCT3(1:1000) as the primary antibody. β‐actin was used as loading control. The lysate of mouse brain and primary neuron were in lane 4 and lane 5 under normal condition. We observed that RCAN1.1 L was the dominant isoform in mouse brain and primary neuron, compared to RCAN1.1S. (F) The protein levels of RCAN1.1 L were determined in the brain tissues from the MCAO group and the Sham group. (G, H) C57BL/6 male mice of 8‐week‐old were performed with the right common carotid artery ligation (R‐CCAL) for different times, which was 0, 2, 4 and 6 h (the ischemic region). The left common carotid artery of each mouse was stimulated, but not ligated (the contralateral region). The RCAN1.1 mRNA and RCAN1.1 L protein levels of the hippocampal CA1 regions of mice were detected by RT‐qPCR and western blotting; 18S was used as loading control for RT‐qPCR and β‐actin was used as loading control for western blotting. (I, J, K, L) Primary neurons (I, J) and SH‐SY5Y (K, L) were treated by OGD for indicated time (0–10 h). RCAN1.1 mRNA and RCAN1.1 L protein were detected by RT‐qPCR and western blotting after OGD treatment. Data are presented as means ± SEM (*n* = 3); *p < 0.05, as calculated by Student's *t* test (for C and F) or one‐way anova with Bonferronis multiple comparisons post hoc test (for G, H, I, J, K and L).

RCAN1.1 has been implicated to be increased in the cortex and hippocampus of Down syndrome and Alzheimer's Disease, leading to neural dysfunction and death.^14^, ^27^, ^28^ However, the expression and function of RCAN1.1 in AIS has not been characterized in detail. To investigate the time‐course of RCAN1.1 expression in AIS in vivo, the ischemic mice model of MCAO was performed. To confirm the MCAO model of AIS, the laser speckle contrast imaging (LSCI) and TTC staining were conducted as shown in Figure 1A,B. The results showed that *RCAN1.1* mRNA in the group of MCAO was sharply increased to 933.7% ± 71.4% of the sham group (*p* = 0.0003) (Figure 1C). As described previously, there are two different isoforms of RCAN1.1 as a consequence of two distinct translational start codons (AUG), a full‐length isoform (RCAN1.1 L) and a shorter isoform (RCAN1.1S).^8^ We found that RCAN1.1 was mainly expressed in neurons in the mice brain (Figure 1D) and RCAN1.1 L, the full‐length isoform, was the major isoform both in the mice brain and primary neurons (Figure 1E). The representative MAP2 immunofluorescence of primary neurons was shown in Figure 6E. Then, we determined the protein level of RCAN1.1 L in the MCAO model of AIS. Consistent with *RCAN1.1* mRNA, the protein level of RCAN1.1 L in the group of MCAO was markedly elevated to 332.5% ± 61.3% of the sham group (*p* = 0.0192) (Figure 1F), indicating that the expression of RCAN1.1 is associated with AIS. To further confirm the consolidation in vivo, the mice model of right common carotid artery ligation (R‐CCAL) was also performed. Similar results were obtained as the MCAO model of AIS. The results showed that *RCAN1.1* mRNA in the right hippocampal CA1 regions (the ischemic region) were sharply increased and peaked up to 1015.0% ± 77.3% at 2 h (*p* < 0.0001), while, a marked reduction in the left hippocampal CA1 regions (the contralateral region) was found (Figure 1G). Then, we determined the protein level of RCAN1.1 L in the mice model of R‐CCAL. Consistent with *RCAN1.1* mRNA, RCAN1.1 L protein levels were markedly elevated in the ischemic regions (peaked up to 1144.0% ± 170.6% at 2 h, *p* = 0.0002), while it was greatly decreased in the contralateral regions (Figure 1H), further indicating that RCAN1.1 may be involved in AIS.

To further confirm the RCAN1.1 expression in AIS progression in vitro, the cellular model of OGD in primary neurons and SH‐SY5Y cells was performed. The results showed that *RCAN1.1* mRNA rapidly elevated at the onset of OGD in the primary neurons and peaked up to 1776.0% ± 125.1% at OGD 6 h (*p* < 0.0001) (Figure 1I). Similar results were obtained in the SH‐SY5Y cells. In SH‐SY5Y cells, *RCAN1.1* mRNA peaked up to 737.9% ± 57.4% at OGD4h (*p* < 0.0001) and persisted until OGD 8 h (Figure 1K). Consistently, the treatment of OGD also upregulated the protein expression of RCAN1.1 L in the primary neurons (Figure 1J) and SH‐SY5Y cells (Figure 1L). The RCAN1.1 L protein peaked up to 1530.0% ± 10.9% at OGD 6 h (*p* = 0.0002) in primary neurons (Figure 1J) and that peaked up to 872.0% ± 102.1% at OGD 8 h (*p* < 0.0001) in SY5Y cells (Figure 1L). Taken together, these results demonstrated a significant upregulation of RCAN1.1 in AIS in vivo and in vitro.

### 3.2 HIF1α activates the *RCAN1.1* promoter

**FIGURE 2:**
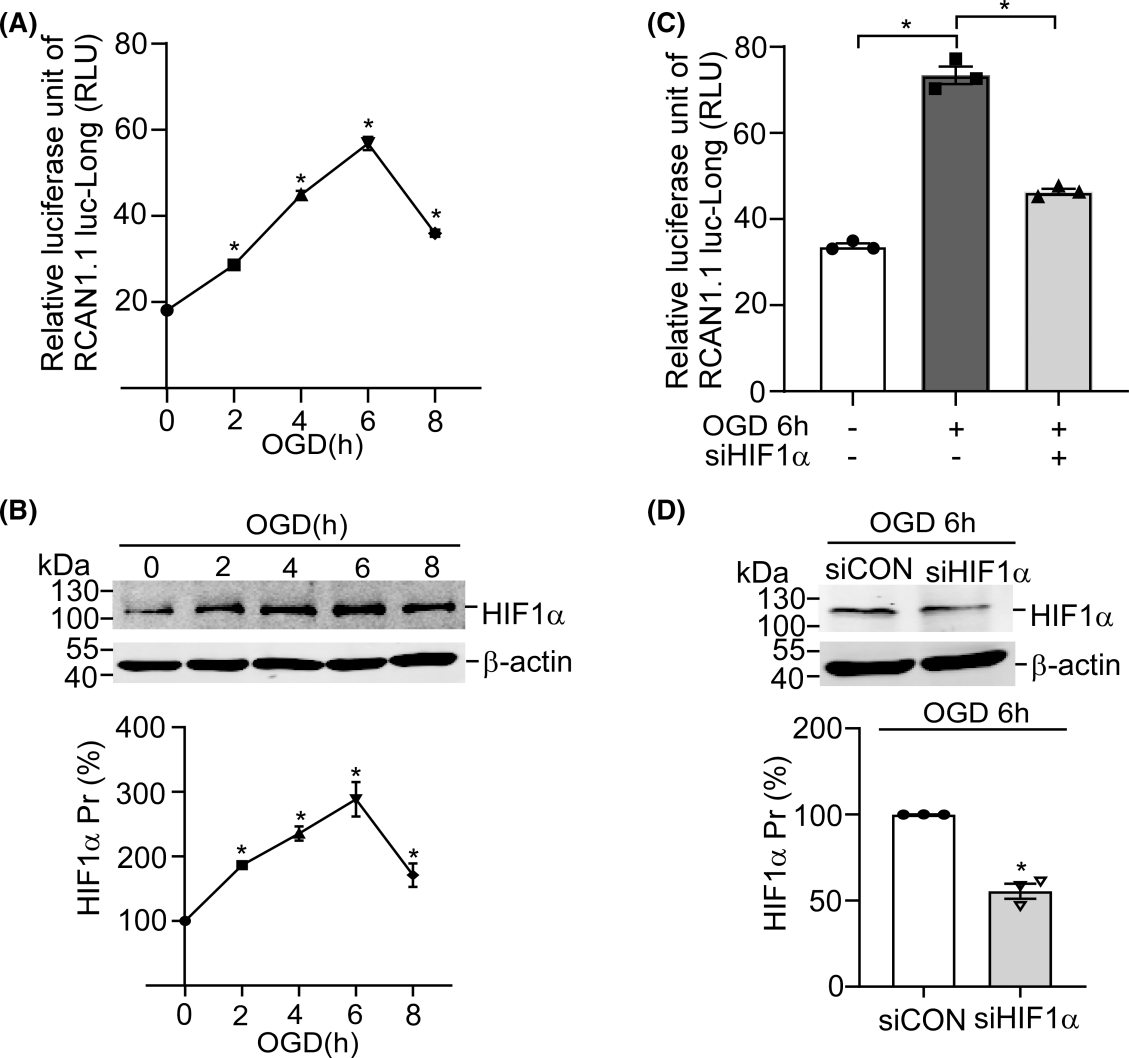
HIF1α activates the RCAN1.1 promoter (A) The RCAN1.1 promoter construct pRCAN1.1 luc‐Long, containing the 5′‐UTR fragment from the human RCAN1.1, was transfected into SH‐SY5Y cells. Cells performed for the indicated time of OGD after 48 h transfection and the dual luciferase activity was measured by a luminometer. (B) SH‐SY5Y cells were performed for the indicated time of OGD and HIF1α protein level was examined by western blotting, β‐actin was used as loading control. (C) The RCAN1.1 promoter construct pRCAN1.1 luc‐Long was co‐transfected with siHIF1α or negative control into SH‐SY5Y cells; 48 h after transfection, cells were performed with or without OGD 6 h and the dual luciferase activity was measured by a luminometer. (D) SH‐SY5Y cells transfected with siHIF1α or negative control (siCON) were harvested 48 h after transfection to detect the protein level of HIF1α by western blotting. β‐actin was used as loading control. Data are presented as means ± SEM (*n* = 3); *p < 0.05, as calculated by Student's *t* test (for D) or one‐way anova with Bonferronis multiple comparisons post hoc test (for A, B and C).

To clarify the underlying molecular mechanism of *RCAN1.1* gene transcription during ischemic stroke, we cloned 1699‐bp fragment (−2000 to −302 bp, *RCAN1.1* translational start codons AUG was used as +1) of the 5′‐UTR region of the human *RCAN1.1* gene into pGL3‐basic plasmid in front of the luciferase reporter gene (Luc), named as pRCAN1.1 luc‐Long. SH‐SY5Y cells transfected with pRCAN1.1 luc‐Long were performed with indicated time of OGD(0–8 h). Dual luciferase assay showed that the relative luciferase activity (RLU) of pRCAN1.1 luc‐Long was markedly upregulated in the course of OGD (Figure 2A, from 18.1 to 45.0 RLU at OGD 4 h, *p* = 0.0096; from 18.1 to 56.8 RLU at OGD 6 h, *p* = 0.0045; from 18.1 to 36.0 RLU at OGD 8 h, *p* = 0.0086; respectively). HIF1α protein was also increased by OGD treatment, consistent with the result of Dual luciferase assay (Figure 2B). To investigate if HIF1α protein affect the *RCAN1.1* promoter, pRCAN1.1 luc‐Long was co‐transfected into SH‐SY5Y cells with siHIF1α to knock down the expression of HIF1α. Dual luciferase assay was performed with or without OGD 6 h after 48 h transfection. The results showed that the RLU of pRCAN1.1 luc‐Long was increased from 33.8 RLU to 73.4 RLU after OGD 6 h treatment (Figure 2C, *p* = 0.0029), while siHIF1α reversely decreased the RLU of pRCAN1.1 luc‐Long to 46.3 RLU compared to siCON (Figure 2C, *p* = 0.0086). The knockdown of siHIF1α was confirmed by western blotting (Figure 2D, *p* = 0.0096). The above results suggest that HIF1α activates the *RCAN1.1* promoter under OGD condition.

### 3.3 Identification of the HRE site in the *RCAN1.1* promoter

**FIGURE 3:**
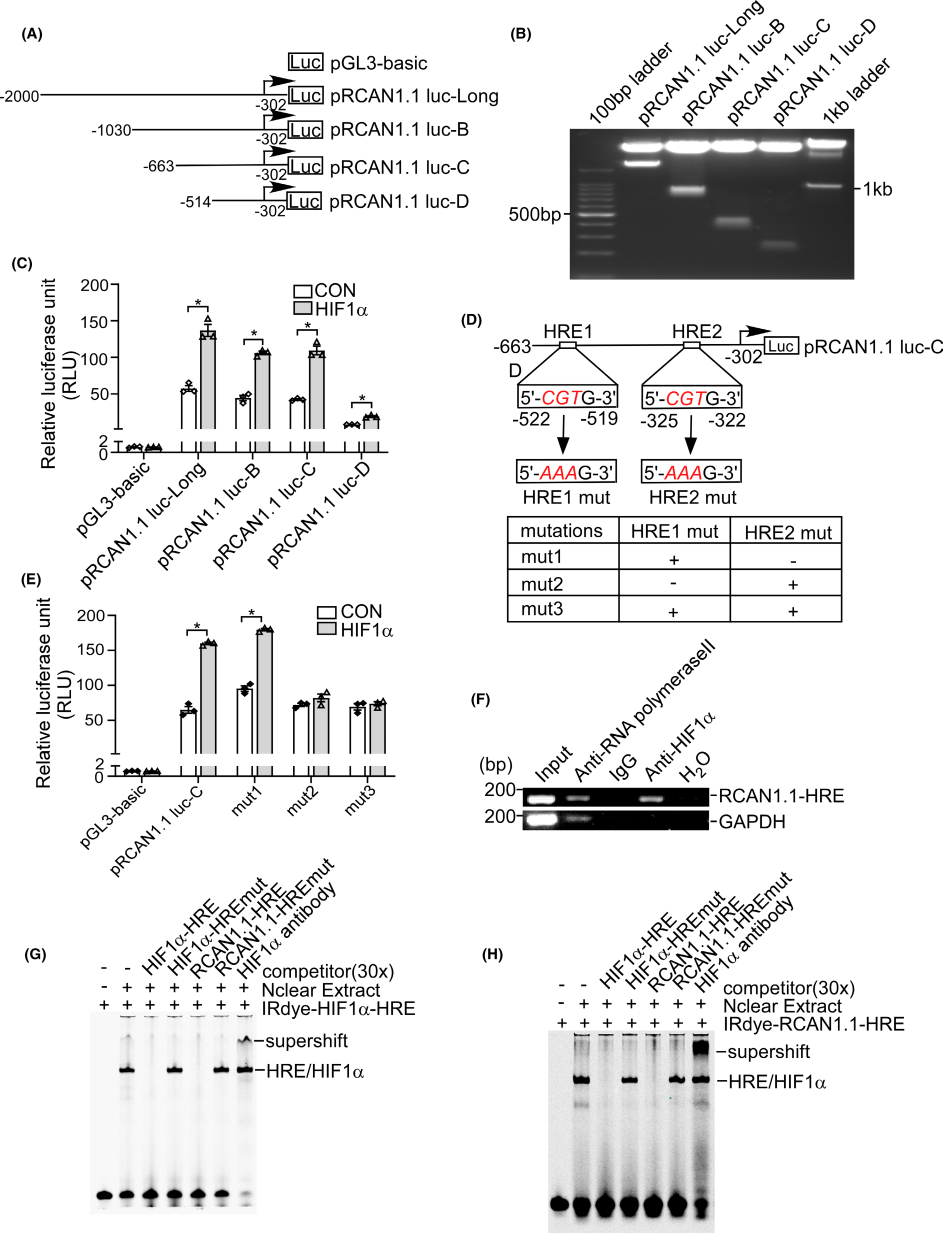
Identification of the hypoxia‐responsive element (HRE) in the RCAN1.1 promoter. (A) Schematic diagrams of the human RCAN1.1 promoter truncation constructs. Four different lengths of 5′ ‐flanking region of the human RCAN1.1 promoter fragment were cloned into pGL3‐basic plasmid in front of the luciferase reporter gene (Luc). Arrow indicates the direction of transcription. The numbers represent the end points of each construct. (B) Sequencing and restriction enzyme digestion assay were performed to confirm all the constructs. The digested products were separated on a 1.5% agarose gel. The vector size is 4.7 kb, and the inserted 5′ ‐flanking fragments of the RCAN1.1 gene ranged from 0.2 to 1.7 kb. (C) RCAN1.1 promoter‐truncation constructs were transfected into SH‐SY5Y cells with either HIF1α expression plasmid (pHA‐HIF1α) or empty vector. Dual luciferase assay was performed at 48 h after transfection; pGL3‐basic was used as the negative control. The dual luciferase activity was measured by a luminometer. (D) The positions of two putative HRE sites are shown at RCAN1.1 promoter region and mutations were constructed by substitution of HRE wild‐type sequence by HRE mutant sequence as indicated. (E) HIF1α expression vector was co‐transfected with pRCAN1.1 luc‐C or pRCAN1.1 luc‐C mut1/2/3 into SH‐SY5Y cells. Dual luciferase assay was performed 48 h after transfection. pGL3‐basic was used as the negative control. (F) The cross‐linked HIF1α‐DNA complex was immunoprecipitated by anti‐HIF1α antibody in CHIP assay. Primers targeting the putative RCAN1.1‐HRE and GAPDH were used for PCR. IgG and H_2_O were used as the negative controls. Anti‐RNA Polymerase II antibody was used as positive control. (G, H) EMSA was performed with IRDye800‐labeled probes as described in Methods. The consensus HRE oligonucleotides (HIF1α‐HRE) and RCAN1.1‐HRE were labeled with IRDye800 and used as probes (IRDye‐HIF1α‐HRE and IRDye‐RCAN1.1‐HRE). Numbers of competitors indicate the molar excess of labeled oligonucleotides. Supershift was carried out with anti‐HIF1α antibody. Data are presented as means ± SEM (*n* = 3); *p < 0.05, as calculated two‐way anova with Bonferronis multiple comparisons post hoc test.

HIF1α protein is a member of Hypoxia‐inducible factor 1 (HIF1) transcription factor family and regulates its target genes through binding to the hypoxia‐responsive element (HRE).^29^ To further clarify the location of the HRE in the *RCAN1.1* promoter, a series of truncation plasmids were constructed by deletion of pRCAN1.1 luc‐Long as follows: pRCAN1.1 luc‐B (−1030 to −302 bp), pRCAN1.1 luc‐C (−663 to −302 bp), pRCAN1.1 luc‐D (−514 to −302 bp) (Figure 3A,B). Each deletion construct was transfected into SH‐SY5Y cells with HIF1α expression plasmid, respectively. The negative control was the empty vector. Dual‐luciferase assay showed that HIF1α significantly increased *RCAN1.1* promoter activity of pRCAN1.1 luc‐Long, pRCAN1.1 luc‐B, pRCAN1.1 luc‐C and pRCAN1.1 luc‐D (Figure 3C), indicating that the region (−514 to −302 bp) of *RCAN1.1* promoter might contain the specific HRE site. A computer‐based sequence analysis of −663 to −302 bp fragment in *RCAN1.1* promoter region showed that there were two putative HRE sites: HRE1(−522 to −519 bp) and HRE2 (−325 to −322 bp). To confirm the conclusion, three mutations of pRCAN1.1 luc‐C were constructed by substitution of HRE wild‐type sequence by HRE mutant sequence as indicated (Figure 3D). Compared with empty vector, the RLUs of pRCAN1.1 luc‐C and pRCAN1.1 luc‐C mut1, but not pRCAN1.1 luc‐C mut2 or pRCAN1.1 luc‐C mut3, were significantly elevated by HIF1α (Figure 3E). These results demonstrated that HRE2 (−325 to −322 bp) is the functional HRE site.

To verify if HIF1α binds to the putative HRE site in the *RCAN1.1* promoter, Chromatin immunoprecipitation (ChIP) assay was employed. The cross‐linked HIF1α‐DNA complex was immunoprecipitated by anti‐HIF1α antibody in CHIP assay. IgG and H_2_O were used as the negative controls. Anti‐RNA Polymerase II antibody was used as positive control. ChIP‐PCR results showed that RCAN1.1‐HRE fragment can be amplified in the presence of HIF1α antibody and anti‐RNA Polymerase II, but not in IgG antibody or H_2_O (Figure 3F).

Electrophoretic mobility shift assay (EMSA) was conducted to investigate whether HIF1α binds to this functional HRE of *RCAN1.1* promoter. The consensus HIF1α‐HRE oligonucleotides were synthesized and labeled with IRDye800 (IRDye‐HIF1α‐HRE), shown as free probe band on DNA polyacrylamide gel electrophoresis (PAGE) gel (Figure 3G, lane 1). A shifted HIF1α/HRE complex band was detected after incubating the IRDye‐HIF1α‐HRE probe with nuclear extract of SH‐SY5Y cells transfected with HIF1α expression plasmid (Figure 3G, lane 2). Unlabeled consensus HRE oligonucleotides and unlabeled mutant consensus HRE oligonucleotides were performed to ensure the specificity of the HIF1α/HRE complex. As expected, unlabeled consensus HRE oligonucleotides (HIF1α‐HRE) with the 30‐fold concentration of labeled probe successfully competed shifted band (Figure 3G, lane 3), whereas mutant consensus HRE oligonucleotides (HIF1α‐HREmut) failed to do so (Figure 3G, lane 4). To confirm the binding of HIF1α and this putative HRE from *RCAN1.1* gene promoter, unlabeled oligonucleotides of RCAN1.1‐HRE and mutant RCAN1.1‐HRE (RCAN1.1‐HREmut) were conducted with 30‐fold concentration of labeled probe. The results suggested that RCAN1.1‐HRE, but not RCAN1.1‐HREmut, could successfully outcompete the shifted HIF1α/ HRE complex band (Figure 3G, lane 5, lane 6). Anti‐ HIF1α antibody was conducted to further confirm the specific binding of HIF1α/ HRE complex (Figure 3G, lane 7).

Similar results were obtained when using the IRDye800‐labeled RCAN1.1‐HRE oligonucleotides as a probe (Figure 3H). The probe also showed a shifted band after incubation with SH‐SY5Y nuclear extract (Figure 3H, lane 2). The intensity of the shifted band was markedly reduced after the addition of unlabeled consensus HRE oligonucleotides and RCAN1.1‐HRE oligonucleotides (Figure 3H, lane 3 and lane 5), while, the mutant consensus HRE or mutant RCAN‐HRE oligonucleotides cannot outcompete the shifted band (Figure 3H, lane 4 and 6). These results demonstrate that HIF1α binds to the specific HRE site (−325 to −322 bp) on the *RCAN1.1* promoter region.

Together, the above results clearly confirm that HIF1α protein activates the *RCAN1.1* promoter and binds to the RCAN1.1‐HRE site at −325 to −322 bp in the *RCAN1.1* promoter region.

### 3.4 RCAN1.1 L facilitates OGD‐induced neuronal apoptosis

**FIGURE 4:**
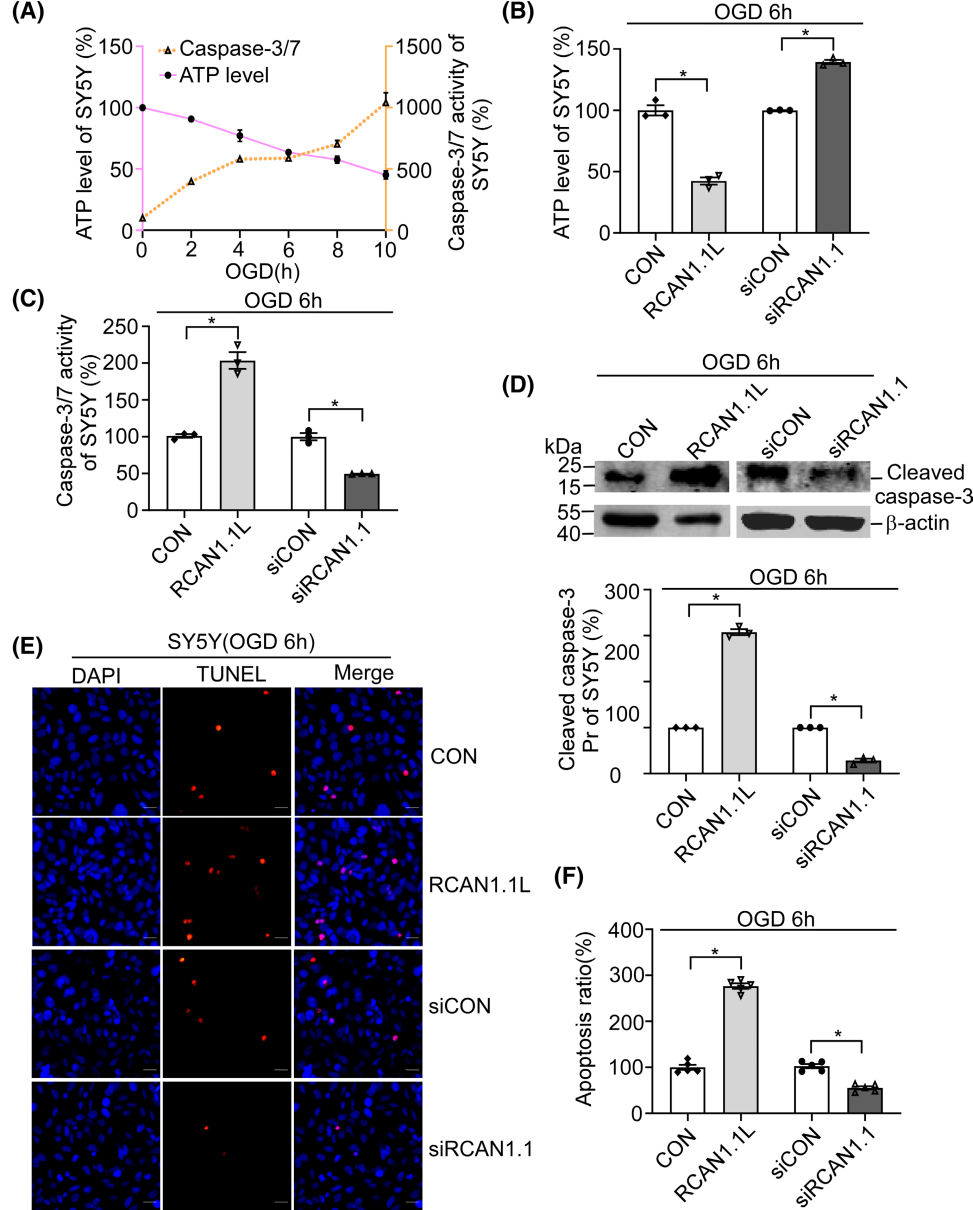
RCAN1.1 L facilitates OGD‐induced apoptosis in the SH‐SY5Y cells. (A) ATP levels (orange) and caspase‐3/7 activities (purple) were detected in the SH‐SY5Y cells under OGD conditions (0, 2, 4, 6, 8 and 10 h). (B, C, D) SH‐SY5Y cells were infected with Lentivirus‐RCAN1.1 L‐GFP (RCAN1.1 L), Lentivirus‐siRCAN1.1‐GFP (siRCAN1.1) and their control viruses for 5 days, respectively (MOI = 1). Cells were harvested after OGD 6 h to detect ATP levels (B) and caspase‐3/7 activities (C). Cleaved caspase‐3 were detected by western blotting. β‐actin was used as loading control. (E, F) SH‐SY5Y cells infected with lentiviruses expressing RCAN1.1 L or siRCAN1.1 for 5 days were performed with TUNEL staining (red) after OGD 6 h treatment. DAPI (blue) was used to indicate the nucleus. The images were captured by a fluorescent microscope. Scale bar 20 μm. F is the quantification of E. Data are presented as means ± SEM (*n* = 3); *p < 0.05, as calculated by one‐way anova with Bonferronis multiple comparisons post hoc test.

Our previous reports showed that in AD and DS patients' brains, RCAN1.1 is overexpressed and induce neuronal apoptosis.^14^ To verify if RCAN1.1 L could affect cell apoptosis under OGD condition, we first detected the time course of ATP levels and caspase‐3/7 activities in SH‐SY5Y cells under different OGD time (OGD 0, 2, 4, 6, 8 and 10 h). The results showed that the ATP levels were sharply decreased in a time‐dependent manner with the OGD time, while caspase‐3/7 activity was significantly increased in SH‐SY5Y cells (Figure 4A). Overexpression of RCAN1.1 L markedly downregulated ATP level to 42.4% ± 2.9% and elevated caspase‐3/7 activity to 203.5% ± 11.0% when compared with their respective control group after OGD 6 h treatment, indicating RCAN1.1 L reduced cell viability during OGD (Figure 4B,C, *p* = 0.0025 and *p* = 0.0114, respectively). Similar conclusion was obtained when the knockdown of RCAN1.1 was applied (Figure 4B,C). Knockdown of RCAN1.1 increased ATP level to 139.4% ± 1.7% and decreased caspase‐3/7 activity to 49.5% ± 4.6% when compared with the respective control group (Figure 4B,C, *p* = 0.0020 and *p* = 0.0082, respectively). The cleaved caspase‐3 protein was also examined and found that RCAN1.1 L sharply increased the cleaved caspase‐3 protein to 307.6% ± 6.5% of CON while on the contrary, a significant downregulation of the cleaved caspase‐3 protein was observed when knockdown of RCAN1.1(Figure 4D, *p* = 0.0010 and *p* = 0.0035, respectively). To further confirm the effect of RCAN1.1 L to cell viability, TUNEL staining were performed to indicate cell apoptosis. Results showed that the RCAN1.1 L‐infected SH‐SY5Y cells exhibited a progressively increased percentage of TUNEL positive cells (276.4% ± 7.4% of CON) after OGD treatment, while on the contrary, a significant reduction of TUNEL positive cells (55.3% ± 3.3% of siCON) was observed when knockdown of RCAN1.1 (Figure 4E,F, *p* < 0.0001 and *p* = 0.0001, respectively). These results demonstrated that RCAN1.1 L could facilitate cell apoptosis under OGD condition in SH‐SY5Y cells.

**FIGURE 5:**
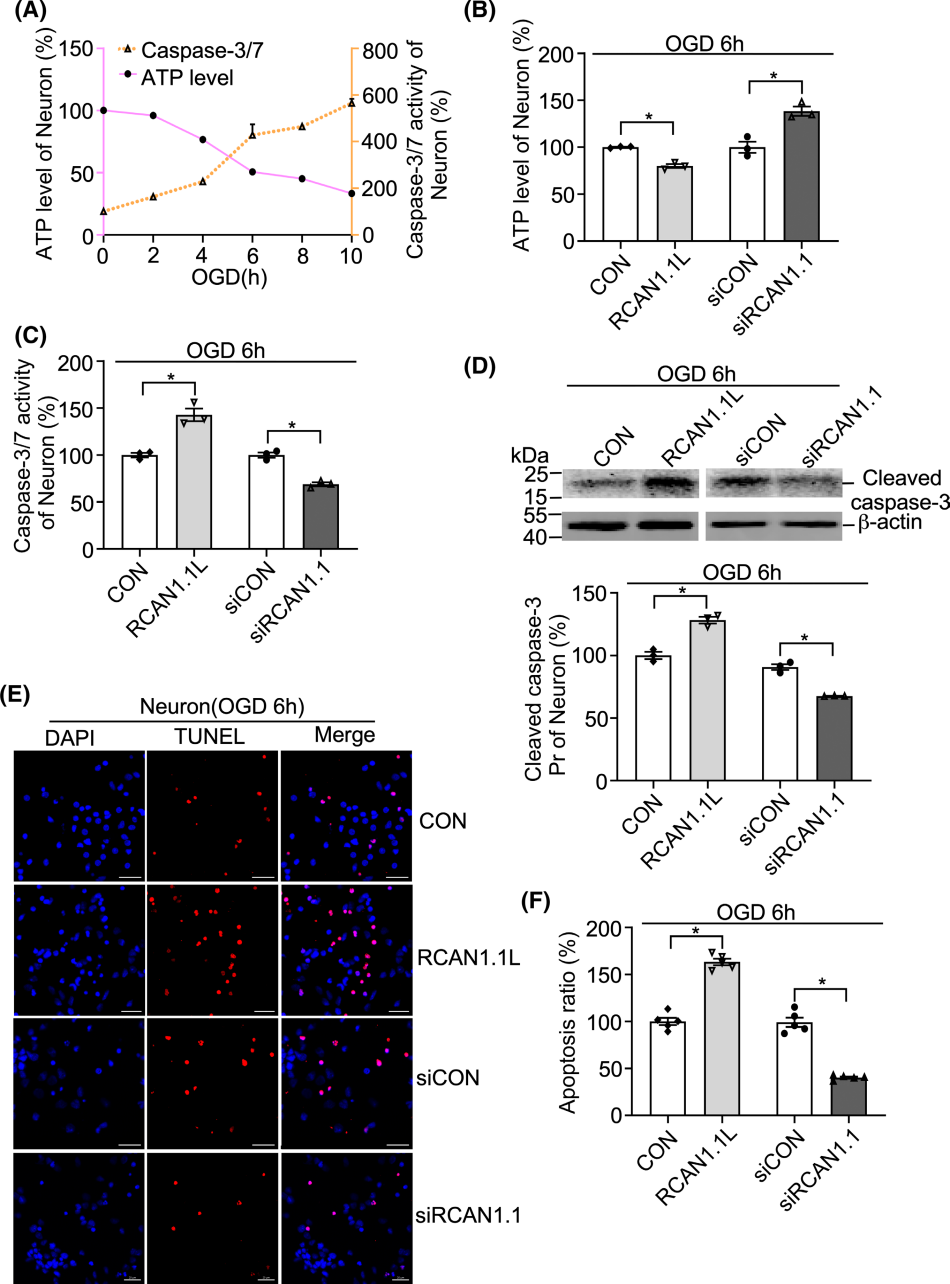
RCAN1.1 L facilitates OGD‐induced apoptosis in primary neurons. (A) ATP levels (orange) and caspase‐3/7 activities (purple) were detected in primary neuron under OGD conditions (0, 2, 4, 6, 8 and 10 h). (B, C, D) Primary neurons were infected with Lentivirus‐RCAN1.1 L‐GFP (RCAN1.1 L), Lentivirus‐siRCAN1.1‐GFP (siRCAN1.1) and their control viruses for 5 days, respectively (MOI = 1). Primary neurons were harvested after OGD6h to detect ATP levels (B) and caspase‐3/7 activities (C). Cleaved caspase‐3 were detected by western blotting. β‐actin was used as loading control. (E, F) Primary neurons infected with Lentiviruses expressing RCAN1.1 L or siRCAN1.1 for 5 days were performed with TUNEL staining (red) after OGD6h treatment. DAPI (blue) was used to indicate the nucleus. The images were captured by a fluorescent microscope. Scale bar was 20 μm. F is the quantification of E. Data are presented as means ± SEM (*n* = 3); *p < 0.05, as calculated by one‐way anova with Bonferronis multiple comparisons post hoc test.

To further investigate the effect of RCAN1.1 L on neuronal apoptosis under OGD condition, the primary neurons were also performed with OGD for different time (OGD 0, 2, 4, 6, 8 and 10 h). Similar results were obtained in primary neurons about the ATP levels and caspase‐3/7 activity in different OGD times (Figure 5A). RCAN1.1 L‐infected primary neurons exhibited a marked downregulation of ATP levels and upregulation of caspase‐3/7 activity under OGD condition, while knockdown of RCAN1.1 resulted in an opposite consequence (Figure 5B,C). The knockdown efficiency of siRCAN1.1 was confirmed by western blotting (Figure S1). The cleaved caspase‐3 protein was sharply increased to 128.3% ± 3.4% by RCAN1.1 L and decreased to 67.6% ± 2.4% by siRCAN1.1 in primary neurons when compared to their respective control (Figure 5D, *p* = 0.0144 and *p* = 0.0102, respectively). The TUNEL staining further demonstrated that excessive apoptosis of primary neurons was triggered by RCAN1.1 L to 163.4% ± 6.7% of CON after OGD, while reduced to 40.2% ± 5.1% of siCON by siRCAN1.1 (Figure 5E,F, *p* = 0.0007 and *p* = 0.0003, respectively). Overall, these results clearly demonstrate that the upregulation of RCAN1.1 is involved in facilitating neuronal apoptosis under OGD conditions.

### 3.5 RISRl3 alleviated RCAN1.1 L‐induced neuronal apoptosis under OGD condition

**FIGURE 6:**
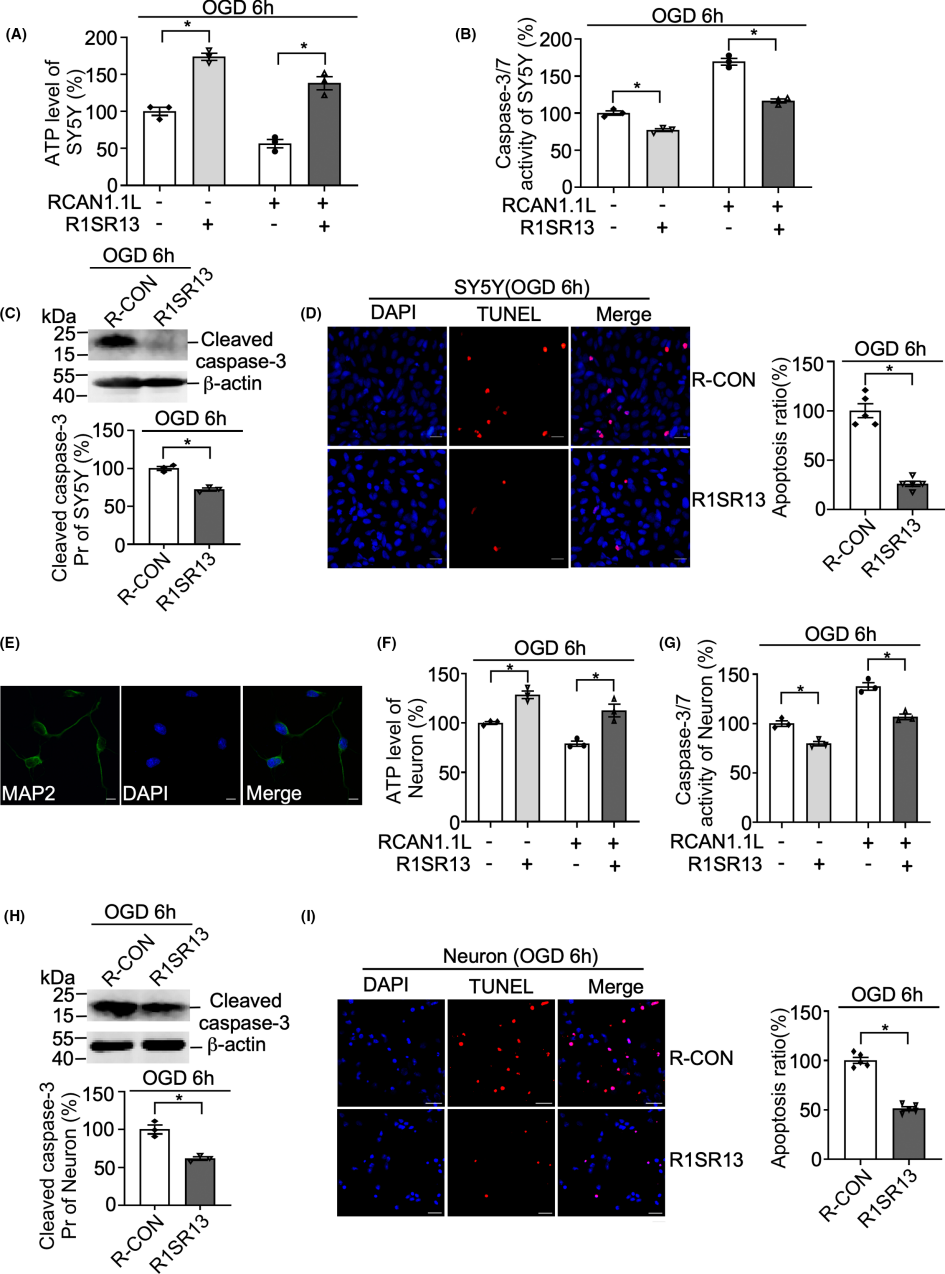
R1SR13 protects SH‐SY5Y cells and primary neurons from apoptosis induced by RCAN1.1 L under OGD conditions. (A, B, F, G) SH‐SY5Y cells (A, B) and primary neurons (F, G) were infected with Lentiviruses expressing RCAN1.1 L or R1SR13 for 5 days. Cells were harvested after OGD 6 h to detect ATP levels and caspase‐3/7 activities. (C, H) Cleaved caspase‐3 protein was detected in the R1SR13‐infected SH‐SY5Y cells (C) and primary neurons (H) after OGD 6 h treatment by western blotting. β‐actin was used as loading control. (E) Representative immunofluorescence images of MAP2 (a specific marker of neuron) in cultured primary neurons. Cultured primary neurons were stained with an anti‐MAP2 (CoraLite488, green) antibody, and DAPI (blue) was used to stain the nucleus. Images were captured with an LSM880 fluorescence confocal microscope. Scale bar was 20 μm. (D, I) TUNEL staining (red) were performed to indicate cell apoptosis in R1SR13‐infected SH‐SY5Y cells (D) and primary neurons (I). DAPI (blue) was used to indicate the nucleus. The images were captured by a fluorescent microscope. Scale bar was 20 μm. Data are presented as means ± SEM (*n* = 3); *p < 0.05, as calculated by Student's *t* test (for C, D, H and I) or one‐way anova with Bonferronis multiple comparisons post hoc test (for A, B, F and G).

Our previous study has demonstrated that RCAN1.1 is a novel RNA‐binding protein and R1SR13 is the RNA aptamer of RCAN1.1 which can block RCAN 1.1‐induced inhibition of the nuclear factor of activated T cells (NFAT)and NFκB signaling pathways.^19^ To examine if R1SR13 could affect neuronal viability under OGD condition, ATP levels, caspase‐3/7 activities, the cleaved caspase‐3 protein and TUNEL staining were performed in SH‐SY5Y cells and primary neurons infected with lentiviruses expressing RCAN1.1 L, R1SR13 aptamer and their negative control, respectively. The representative MAP2 immunofluorescence of primary neuron was shown in Figure 6E. Consistent with previous results, overexpression of RCAN1.1 L markedly reduced the cell viability indicated by decreased ATP levels and increased caspase‐3/7 activities, while co‐expression of R1SR13 with RCAN1.1 L restored the cell viability under OGD condition (Figure 6A,B,F,G). Furthermore, the cleaved caspase‐3 protein was significantly downregulated by R1SRl3 compared with negative control in SH‐SY5Y cells and primary neurons (Figure 6C,H). To consolidate the notion, TUNEL staining was also performed, and it was found that the RNA aptamer R1SR13 sharply reduced the percentage of TUNEL positive cells induced by RCAN1.1 L (Figure 6D,I). Together, these results clearly confirm that RISRl3 can inhibit RCAN1.1 L‐induced neuronal apoptosis under OGD condition.

### 3.6 R1SR13 alleviated brain injury in AIS via inhibiting neuronal apoptosis in vivo

**FIGURE 7:**
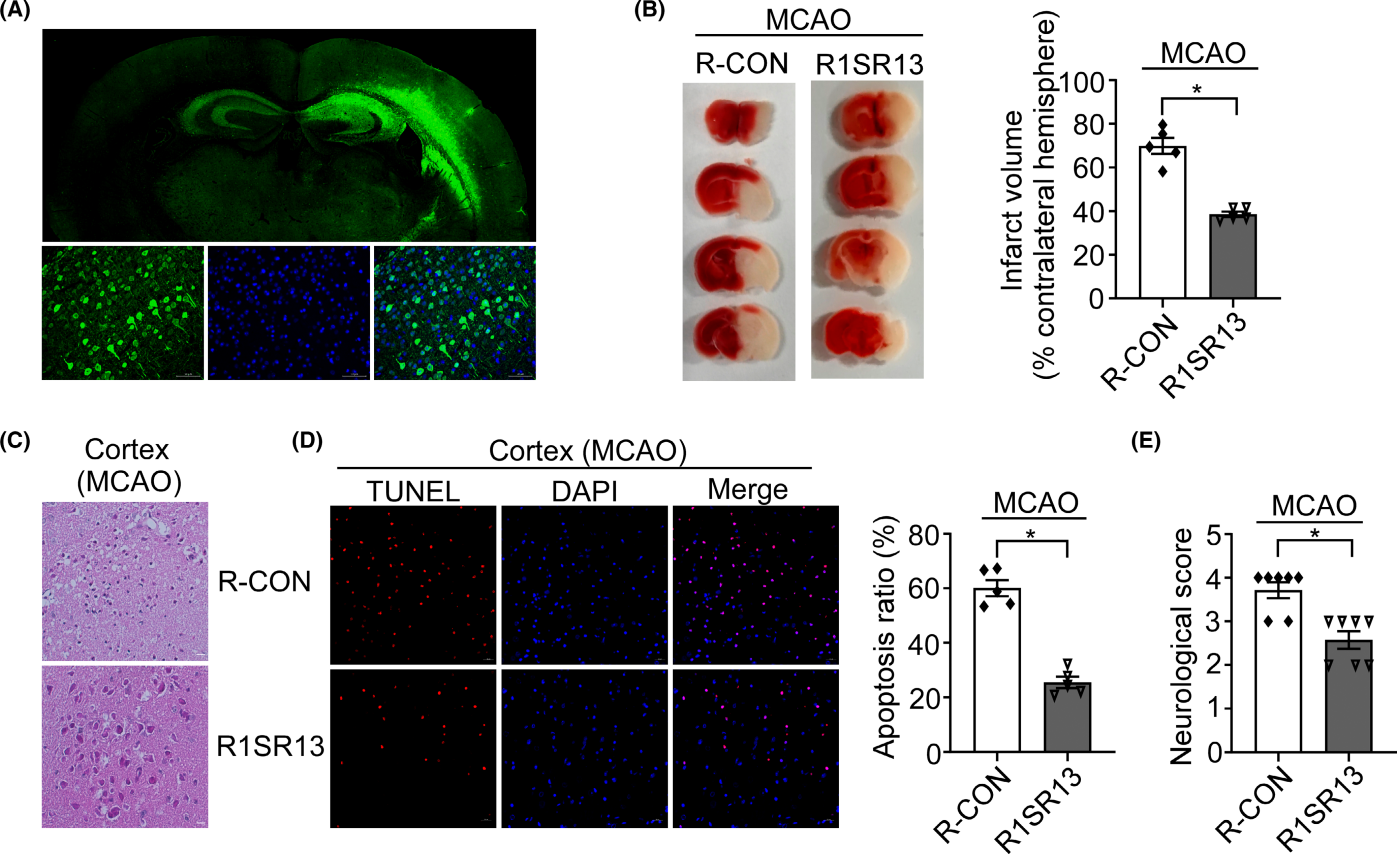
R1SR13 alleviated brain injury in AIS via inhibiting neuron apoptosis in vivo. Adeno‐associated virus 9 (AAV9) containing R1SR13 or the control virus was injected into the right brain of the male C57BL/6 mice and the mice were subjected to MCAO surgery 3 weeks after viral infection. (A) The AAV9‐R1SR13 efficiently infected mouse brain. (B) The infarct volume was evaluated by TTC staining after 24 h of MCAO. (C) Representative photomicrographs of HE staining in the cortex. Scale bar 20 μm. (D) Representative images and quantification of apoptosis by TUNEL staining (red) in the ischemic brain 24 h after MCAO. DAPI (blue) was used to indicate the nucleus. Scale bar was 20 μm. (E) Neurological deficit scores were evaluated after 24 h of MCAO from different group. Data are presented as means ± SEM (*n* = 5); *p < 0.05, as calculated by Student's *t* test.

To further confirm the functional role of R1SR13 in AIS in vivo, we injected AAV9‐R1SR13/AAV9‐R‐CON into the right brain of the mice by the stereotaxic apparatus. The efficiency of the injection was evaluated by GFP fluorescence in brain sections. The results showed that the virus effectively diffused in the right brain (Figure 7A). We observed that R1SR13 markedly reduced the infarct size induced by MCAO as shown by TTC staining (Figure 7B, *p* < 0.0001). Furthermore, R1SR13 alleviated cell apoptosis at 24 h after MCAO and neuronal morphological damage in the cortex as shown by TUNEL staining (Figure 7D, *p* < 0.0001) and HE staining (Figure 7C), respectively. In addition, the downregulation of neurological scores was also observed in the group of R1SR13, comparing to the control group (Figure 7E, *p* = 0.0013). Collectively, our results indicated that R1SR13 played a critical role in regulating brain ischemic injury, and thus alleviated the cerebral ischemic injury in vivo.

## 4 DISCUSSION

**FIGURE 8:**
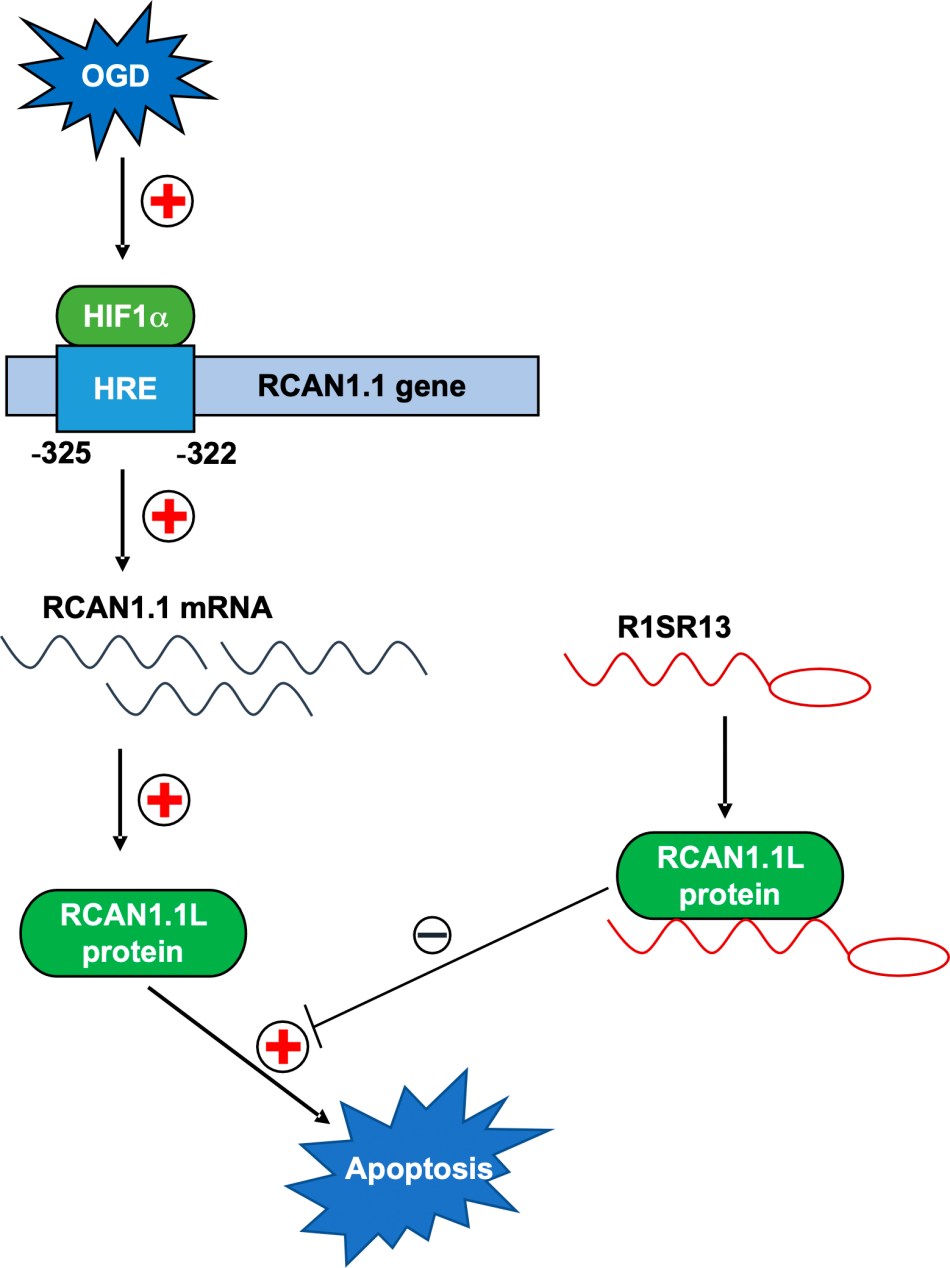
Schematic diagram of upregulated RCAN1.1 L expression by HIF1α in acute ischemic stroke and the protection of R1SR13 in ameliorating cell apoptosis. Activation of OGD results in the upregulation of HIF1α in nucleus and HIF1α interacts with the specific HRE in the *RCAN1.1* promoter, subsequently prompting the expression of RCAN1.1 L protein. The enhanced expression of RCAN1.1 L facilitates the cell apoptosis under OGD conditions, while the addition of R1SR13 ameliorates cell apoptosis through competitively inhibiting the function of RCAN1.1 L protein.

In the current study, we provided evidence for the first time that the RNA aptamer of RCAN1.1, R1SR13, played a neuroprotective role in the cellular and animal models of AIS by competitively inhibiting the pro‐apoptotic effect of the markedly elevated RBP, RCAN1.1, as shown in Figure 8. We proved a significant neuronal upregulation of RCAN1.1 in the cellular and animal models of AIS. Mechanistically, we showed that HIF1α activated the expression of RCAN1.1 through binding to the specific RCAN1.1‐HRE (−325 to −322 bp) in *RCAN1.1* gene promoter region. Furthermore, the overexpression of RCAN1.1 in AIS resulted in elevated neuronal apoptosis indicated by TUNEL, decreased ATP levels, increased caspase3/7 activities and cleaved caspase‐3.

As “chemical antibodies”, aptamers are widely used for targeted therapy because of some advantages, such as lack of immunogenicity, rapid tissue penetration, cell internalization and so on. Several aptamers have been proved to be potential for the targeted therapy of different diseases such as pegaptanib (an VEGF_165_‐inhibiting aptamer) for age‐related macular degeneration, AS1411 for cancer and UCLA1 for viral infection.^30^ Our recent study firstly discovered that RCAN1.1 is a novel RNA binding protein and identified the RNA recognition motif (RRM). The RRM domain is highly conserved and is contained both in RCAN1.1 L and RCAN1.1S isoforms. R1SR13, as the RNA aptamer of RCAN1 identified by SELEX, may compete with the physiological RNAs due to the higher binding affinity to RCAN1 proteins. The binding affinity of R1SR13 with RCAN1 is within nanomolar range that is an approach to the antibody–antigen avidity.^19^ To investigate if R1SR13 could alleviate the RCAN1.1 L‐induced neuronal apoptosis in AIS, the RNA aptamer R1SR13 was applied to SH‐SY5Y cells and primary neurons treated by OGD for indicated time. Results from ATP assays, caspase3/7 assays and TUNEL staining showed that R1SR13 could invert RCAN1.1 L‐induced changes. Moreover, a downregulation of the cleaved caspase‐3 protein was also observed in the lentivirus‐R1SR13‐treated group both in SH‐SY5Y cells and primary neurons under OGD condition. In addition, the ischemic model of MCAO was also performed to further confirm the protective function of R1SR13 in AIS in vivo. Results showed that R1SR13 markedly reduced the infarct size and apoptosis ratio induced by MCAO, as well as neurological scores. These results indicated that the addition of R1SR13 may evoke a protective effect through inhibiting the binding of RCAN1.1 L with other apoptosis‐related mRNAs in AIS.

The above results were based on our discovery that RCAN1.1 was significantly elevated in cellular and animal models of AIS and its pro‐apoptotic effect. According to our previous studies, RCAN1.1 is a multifunctional protein and is enriched in the brain. We verified the significant expression of RCAN1.1 mRNA and protein in two mouse models of MCAO and R‐CCAL. MCAO is the recognized focal model of cerebral ischemia, and R‐CCAL is an anterior circulation ischemic model of the cerebral hemisphere.^31^ Our results were consistent in these two models above, as well as in the SH‐SY5Y cells and primary neurons under OGD conditions.

Why was RCAN1.1 so markedly elevated in neurons of AIS? It is well known that oxygen homeostasis is critical for the development and physiology of an organism. In AIS pathogenesis, OGD is a direct consequence of hypoperfusion. Among the genes regulating oxygen homeostasis, the roles of HIF1 have attracted widespread attention. HIF1α protein is a functional subunit of HIF1 and binding to its target genes with a specific region named HRE.^23^, ^32^ We explored the relationship between RCAN1.1 and HIF1α. The results showed that the change of RCAN1.1 level was due to the activation of the *RCAN1.1* promoter by HIF1α protein under OGD condition. Moreover, we also identified that HIF1α directly binds to the specific RCAN1.1‐HRE (−325 to −322 bp) in *RCAN1.1* gene promoter region.

Apoptosis is the dominating cell death type in the ischemic penumbra and the apoptosis in the first hours after ischemic stroke may be salvaged.^33^, ^34^ ATP production is suppressed after ischemic stroke, and caspase‐3 and caspase‐7 are important executors of apoptosis. ATP levels and capase3/7 activities were widely used to indicate cell viability.^35^, ^36^ In our present study, we found that overexpression of RCAN1.1 could inhibit cell viability indicating by decreased ATP levels and increased capase‐3/7 activities both in SH‐SY5Y cells and primary neurons. Furthermore, a significantly increase of the cleaved caspase‐3 protein was discovered by RCAN1.1, accompanied by more TUNEL‐positive cells. Moreover, to confirm these results, the knockdown of RCAN1.1 was also performed. On the contrary, knockdown of RCAN1.1 led to higher cell viability and less cell apoptosis in OGD‐treated cells. These results suggest that RCAN1.1 facilitates neuronal apoptosis in the AIS progression.

ATP is produced intracellularly by mitochondria, and reduced ATP production implies mitochondrial dysfunction. In our previous study, it was found that RCAN1.1 stabilized and raised the expression of *ANT1* mRNA, through binding to 269–291 nt of *ANT1* mRNA.^19^ ANT1 is the most abundant protein in the inner mitochondrial membrane. It forms as a homodimer, a gated channel by which ADP is brought into and ATP brought out of the mitochondrial matrix. RCAN1.1 interacted with ANT1 in mitochondria and facilitated mPTP opening, resulting in Cyt c release and apoptosis.^15^ Besides, Our RIP‐seq and KEGG pathway analysis also suggested the potential roles of RCAN1.1 in mitochondrial functions.^19^ In addition to *ANT1* mRNA, we found RNA motifs that could bind to RCAN1.1 in vivo‐based on the results of RIP‐seq, and confirmed the high affinity of protein and motifs. However, which mRNA these motifs are located in, and whether they play a role in regulating apoptosis needs to be explored further.

In conclusion, our present study has provided evidence that RCAN1.1 expression is significantly increased in AIS both in vivo and in vitro. HIF1α activates the expression of RCAN1.1 through binding to the region of −325 to −322 bp in the *RCAN1.1* gene promoter. Furthermore, RNA aptamer R1SR13 has a protective role in AIS via alleviating neuronal apoptosis both in vivo and in vitro. Thus, R1SR13 may appear to be a potential therapeutic aptamer for AIS neuronal prevention.

## CONFLICT OF INTEREST

The authors declare no competing financial interests.

## Supporting information


Figure S1

## Data Availability

The data that support the findings of this study are available from the corresponding author upon reasonable requests
