# Supplementary figures and images for: Targeting upregulated RNA binding protein RCAN1.1: a promising strategy for neuroprotection in acute ischemic stroke
Source: CNS Neurosci Ther. 2022 Jul 28;28(11):1814–28. doi: 10.1111/cns.13921 (PMC9532900; doi:10.1111/cns.13921)

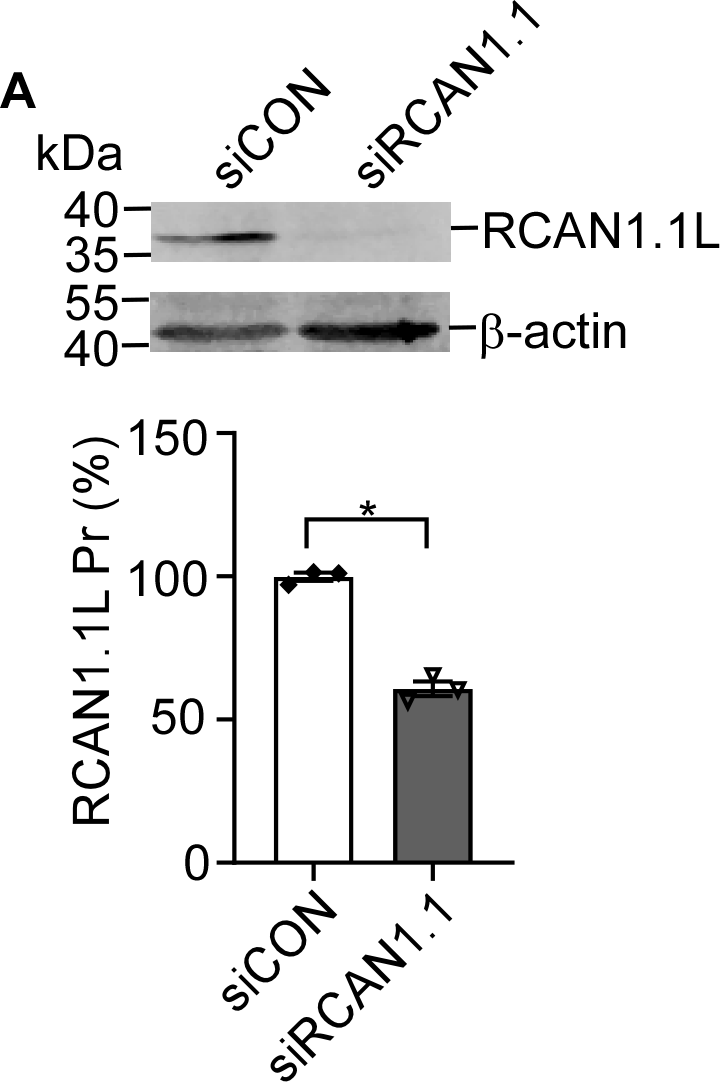

Supplement: Supplementary file 1 — Figure S1 [file CNS-28-1814-s001.tif]
